# Improving the signal subtle feature extraction performance based on dual improved fractal box dimension eigenvectors

**DOI:** 10.1098/rsos.180087

**Published:** 2018-05-02

**Authors:** Xiang Chen, Jingchao Li, Hui Han, Yulong Ying

**Affiliations:** 1 State Key Laboratory of Complex Electromagnetic Environment Effects on Electronics and Information System (CEMEE), Luoyang, Henan 471003, People's Republic of China; 2 College of Electronic and Information Engineering, Shanghai Dianji University, Shanghai 201306, People's Republic of China; 3 School of Energy and Mechanical Engineering, Shanghai University of Electric Power, Shanghai 200090, People's Republic of China

**Keywords:** radiation source signal, subtle features, traditional fractal box-counting dimension, improved fractal box-counting dimension, grey relation algorithm

## Abstract

Because of the limitations of the traditional fractal box-counting dimension algorithm in subtle feature extraction of radiation source signals, a dual improved generalized fractal box-counting dimension eigenvector algorithm is proposed. First, the radiation source signal was preprocessed, and a Hilbert transform was performed to obtain the instantaneous amplitude of the signal. Then, the improved fractal box-counting dimension of the signal instantaneous amplitude was extracted as the first eigenvector. At the same time, the improved fractal box-counting dimension of the signal without the Hilbert transform was extracted as the second eigenvector. Finally, the dual improved fractal box-counting dimension eigenvectors formed the multi-dimensional eigenvectors as signal subtle features, which were used for radiation source signal recognition by the grey relation algorithm. The experimental results show that, compared with the traditional fractal box-counting dimension algorithm and the single improved fractal box-counting dimension algorithm, the proposed dual improved fractal box-counting dimension algorithm can better extract the signal subtle distribution characteristics under different reconstruction phase space, and has a better recognition effect with good real-time performance.

## 1. Introduction

Radiation source signal recognition technology is an emerging field in information warfare. Exploring effective signal feature extraction technology under a lower signal-to-noise ratio and achieving identification of different radiation source signals is the technical basis for the development of a new generation of electronic counterintelligence reconnaissance equipment. Owing to the complexity and urgency of identifying individual radiation sources, this has been a key topic in the field of information warfare. Therefore, it is necessary to develop a reliable and effective signal subtle feature extraction algorithm for radiation source signal recognition. Currently, many signal processing techniques have been applied to signal subtle feature extraction. However, the radiation source signal exhibits nonlinear and unsteady characteristics as a result of many nonlinear factors [1]. Slight radiation source signal information is easily submerged in the inevitable background noise and becomes difficult to extract. Therefore, conventional time-domain and frequency-domain methods may not make an accurate assessment of the radiation source signal subtle feature [2]. With the development of nonlinear dynamics, many nonlinear analytical techniques have been applied to identifying and predicting the complex dynamic nonlinearity of the radiation source signal [3]. Among them, the most typical technique is to extract the characteristic frequency from the radiation source signal through the combined usage of some advanced signal processing techniques (higher order spectra [4], wavelet package transform [5], the Hilbert transform [6], empirical mode decomposition, etc.) and further evaluating the radiation source signal by comparing it with the theoretical characteristic frequency value with the aid of expert judgement. With the development of artificial intelligence [7], radiation source signal recognition technology has been gradually introduced into the category of pattern recognition. And the validity and reliability of radiation source signal recognition technology are mainly determined by the selection of the dominant eigenvectors which characterize the signal subtle features. Recently, some entropy-based methods (hierarchical entropy [8], fuzzy entropy [9], sample entropy [10], approximate entropy [11], hierarchical fuzzy entropy [12], etc.) have been proposed for extracting dominant eigenvectors from a radiation source signal and have achieved some effect.

After extracting signal subtle features, a pattern recognition technique is also needed to achieve the radiation source signal recognition. Nowadays, a variety of pattern recognition methods have been used in radiation source signal recognition, of which the most widely used are support vector machines (SVMs) [13] and artificial neural networks (ANNs) [14–16]. Among them, ANN training requires a large number of samples, which is difficult or even impossible in practical applications. SVMs are based on statistical learning theory (which is especially suitable for training with a small number of samples), and have better generalization ability than ANNs and can ensure that local optimal solutions and global optimal solutions are consistent [17]. However, the accuracy of SVM classifiers depends on the choice of their optimal parameters [18,19]. To ensure the accuracy of radiation source signal recognition, some optimization algorithms and the design of complex multi-class structures [20] are often integrated into SVMs. Here, in order to solve the issue of generality versus accuracy, the grey relation algorithm (GRA) was developed to achieve accurate pattern recognition based on a small number of samples.

Aimed at solving the above problems, a dual improved generalized fractal box-counting dimension eigenvector algorithm is proposed in this paper. To achieve this, first the radiation source signal was preprocessed, and a Hilbert transform was performed to obtain the instantaneous amplitude of the signal. Then the improved fractal box-counting dimension of the signal instantaneous amplitude was extracted as the first eigenvector. At the same time, the improved fractal box-counting dimension of the signal without the Hilbert transform was extracted as the second eigenvector. Finally, the dual improved fractal box-counting dimension eigenvectors formed the multi-dimensional eigenvectors as signal subtle features, which were used for radiation source signal recognition by the GRA.

The rest of the paper is organized as follows. First, the proposed method is introduced in §2. The application and analysis of the proposed method are illustrated in §3. The conclusion is presented in §4.

## 2. Methodology

### 2.1. Hilbert transform

The Hilbert transform is widely used in signal processing to obtain an analytic representation of the signal, which allows instantaneous calculation of the amplitude, phase and frequency.

Given an actual radiation source signal f(t), its Hilbert transform is defined as follows:

2.1 $$h(t)=\frac{1}{\pi }P\int_{-\infty }^{\infty }\frac{f(\tau )}{t-\tau }\text{d}\tau ,$$

where *P* is a Cauchy Lord value.

It can be seen from equation (2.1) that h(t) is a time-dependent linear function. The following relationship can be obtained from f(t) by applying convolution with ${\left(\pi t\right)}^{-1}$:

2.2 $$h(t)=\frac{1}{\pi t}*f(t).$$

Then the Fourier transform can be used to obtain the following equation:

2.3 $$F\{h(t)\}=\frac{1}{\pi }F\left\{\frac{1}{t}\right\}F\{f(t)\}.$$

Since

2.4 $$F\left\{\frac{1}{t}\right\}=\int_{-\infty }^{\infty }\frac{1}{x}{\text{e}}^{-j2\pi fx}\text{d}x=-j\pi \;\text{sgn}\;f,$$

where if f>0, sgn f is +1; if f=0, sgn f is 0; and if f<0, sgn f is −1.

Then the following equation can be obtained:

2.5 F{h(t)}=−j sgn fF{f(t)}.

Then the time-domain result h(t) can be obtained by performing an inverse Fourier transform. The Hilbert transform h(t) of the radiation source signal f(t) represents its harmonic conjugate. The Hilbert transform can be used to obtain an analytic signal z(t) from an actual radiation source signal f(t), and the analytic signal z(t) can be expressed as follows:

2.6 $$z(t)=c(t)+ih(t)=|z(t)|{\text{e}}^{i\theta (t)}.$$

Therefore, the instantaneous amplitude of the analytic signal z(t) is

2.7 $$|z(t)|=\sqrt{f^{2}(t)+h^{2}(t)}.$$

### 2.2. Traditional fractal box-counting dimension

Fractal theory is one of the most important branches of contemporary nonlinear sciences [21], and is suitable for processing all types of nonlinear and non-stationary phenomena; it may also be suitable for subtle feature extraction from radiation source signals. The fractal box-counting dimension algorithm has the advantage of simple calculation compared with other fractal dimension algorithms. The conventional algorithm of the fractal box-counting dimension has been widely used in the fields of image analysis [22], electromagnetic fault diagnosis and biomedicine [23], in which signals are strictly self-similar.

Suppose *A* is a non-empty bounded subset of Euclidean space $R^{n}$ to be calculated, and N(A,ε) is the least number of boxes with the side length ε covering *A*. Then the fractal box-counting dimension can be defined as

2.8 $$D=\underset{\varepsilon \to 0}{\lim}\frac{\log N(A,\varepsilon )}{\log(1/\varepsilon )}.$$

For the actual radiation source signal sequence f(i), $i=1,2,\ldots ,N_{0}$, there is no meaning for ε→0 to calculate the box-counting dimension as the sampling interval σ is the highest resolution of the signal because of the existence of the sampling frequency. Therefore, the minimum side length ε of the box is often said to be equal to σ. Consider the actual sampled radiation source signal sequence f(i) as the closed set of Euclidean space $R^{n}$, and the calculation process of the fractal box-counting dimension is described as follows.

Use the approximate method to make the minimum side length ε of the box covering the radiation source signal sequence f(i) equal to the sampling interval σ. And calculate the least number of boxes N(kε) with side length kε covering the signal sequence f(i), thus

2.9 $$\begin{array}{r} p_{1}=\max\{\;f(k(i-1)+1),f(k(i-1)+2),\ldots ,f(k(i-1)+k+1)\}, \end{array}$$

2.10 $$\begin{array}{r} p_{2}=\min\{\;f(k(i-1)+1),f(k(i-1)+2),\ldots ,f(k(i-1)+k+1)\} \end{array}$$

2.11 $$\begin{array}{r} \text{and}\;\;\;p(k\varepsilon )=\sum_{i=1}^{N_{0}/k}\left|p_{1}-p_{2}\right|, \end{array}$$

where $i=1,2,\ldots ,N_{0}/k$, k=1,2,…,K. $N_{0}$ is the number of sampling points, $K<N_{0}$. p(kε) is the longitudinal coordinate scale of the actual sampled radiation source signal sequence f(i). Thus N(kε) can be defined as

2.12 $$N(k\varepsilon )=\frac{p(k\varepsilon )}{k\varepsilon }+1.$$

Select a fitting curve log⁡kε∼log⁡N(kε) with good linearity as a scale-free zone, and the fitting curve can be defined as

2.13 log⁡N(kε)=alog⁡kε+b,

where $k_{1}\leq k\leq k_{2}$ and $k_{1}$ and $k_{2}$ are the beginning and end of the scale-free zone, respectively.

Generally, a least squares method is used to calculate the slope of the fitting curve, which is the fractal box-counting dimension *D* of the actual sampled radiation source signal sequence f(i),

2.14 $$D=-\frac{(k_{2}-k_{1}+1)\sum \log k\cdot \log N(k\varepsilon )-\sum \log k\cdot \sum \log N(k\varepsilon )}{(k_{2}-k_{1}+1)\sum \log^{2}k-{\left(\sum \log k\right)}^{2}}.$$

### 2.3. Improved generalized fractal box-counting dimension

However, for the actual radiation source signals, they do not satisfy the self-similar structure of fractal theory to some degree. Therefore, when using the traditional fractal box-counting dimension algorithm to calculate the box-counting dimension of the radiation source signals, the fitting curve often does not have a good linear structure. Because of this problem, an improved generalized fractal box-counting dimension algorithm was developed to overcome the defect of the conventional fractal box-counting dimension algorithm. The specific calculation procedure is as follows.

(1) Resample the actual radiation source signal sequence f(i), $i=1,2,\ldots ,N_{0}$, and properly increase the sampling points to reduce the minimum side length *ϵ* to improve the calculation accuracy of the fractal box-counting dimension of the signal sequence f(i). The phase space of the signal sequence f(i) is reconstructed, and the number of iterated dimensions of the reconstructed phase space is determined according to the number of sampling points.
(2) Suppose the number of sampling points of the signal sequence f(i) is $N_{0}=2^{n}$. To improve the calculation accuracy, resample the actual radiation source signal sequence f(i), and suppose the number of sampling points of the signal sequence f(i) is $N=2^{K}$ (K>n). The reconstruction dimension of the phase space of the signal sequence f(i) is set, respectively, as $m=K+1=2,3,4,\ldots ,\log_{2}N+1$.
(3) The derivate process of the number of boxes covering the actual radiation source signal sequence f(i) can be described as follows.
  When k=1:
  $p_{1}=\max\{f(i),f(i+1)\}$, $p_{2}=\min\{f(i),f(i+1)\}$, *i *= 1,2, … , *N*/*k*. In this case, the reconstructed phase space dimension is 2.
  When k=2:
  $p_{1}=\max\{f(2i-2),f(2i),f(2i+1)\}$, $p_{2}=\min\{f(2i-2),f(2i),f(2i+1)\}$, i=1,2,…,N/k. In this case, the reconstructed phase space dimension is 3.
  When k=3:
  $p_{1}=\max\{f(3i-2),f(3i-1),f(3i),f(3i+1)\}$, $p_{2}=\min\{f(3i-2),f(3i-1),f(3i),f(3i+1)\}$, i=1,2,…,N/k. In this case, the reconstructed phase space dimension is 4.
  When k=K:
  $p_{1}=\max\{f(Ki-K+1),f(Ki-K+2),\ldots ,f(Ki+1)\}$, $p_{1}=\min\{\;f(Ki-K+1),f(Ki-K+2),\ldots ,f(Ki+1)\}$, i=1,2,…,N/k. In this case, the reconstructed phase space dimension is *m *= *K *+ 1.
(4) It can be seen from the above deduction that, during the reconstruction of the phase space of the radiation source signal sequence f(i) *K* times, the corresponding log⁡N(kε) can be obtained at each time. And then the relation curve of log⁡kε∼log⁡N(kε) can be drawn. Since the fitting curve does not have a strict linear relationship, take the derivation of the relation curve at these *K* points over the improved generalized fractal box-counting dimension algorithm. The slopes $D_{1},D_{2},D_{3},\ldots ,D_{K}$ at these *K* points from the relation curve are the fractal box-counting dimensions in the different reconstructed phase space. Take the slopes $D_{1},D_{2},D_{3},\ldots ,D_{K}$ obtained as the *K* characteristic parameters for the feature vector extracted from the signal sequence f(i), which characterizes the signal subtle features.

### 2.4. Grey relation theory

The study of grey relation theory is the foundation of grey system theory, which is mainly based on the basic theory of space mathematics, which is used to calculate the relation coefficient and relation degree between the reference characteristic vector and each comparative characteristic vector. Grey relation theory has a good potential to be used in radiation source signal recognition for the following reasons [24]: (i) it has good tolerance to measurement noise; (ii) its algorithm is simple and can solve the issue of generality versus accuracy; (iii) it can solve the learning problem with a small number of samples; and (iv) it has the ability to assist the selection of characteristic parameters for classification.

Suppose the feature vectors $\left[D_{1}^{\prime },D_{2}^{\prime },\ldots ,D_{K}^{\prime },D_{1},D_{2},\ldots ,D_{K}\right]$ (i.e. the dual improved generalized fractal box-counting dimension eigenvectors) extracted from the radiation source signal to be identified are as follows:

2.15 $$B_{1}=\left[\begin{array}{c} b_{1}(1)\\ b_{1}(2)\\ b_{1}(3)\\ \ldots \\ b_{1}(2K) \end{array}\right],\;\;B_{2}=\left[\begin{array}{c} b_{2}(1)\\ b_{2}(2)\\ b_{2}(3)\\ \ldots \\ b_{2}(2K) \end{array}\right],\ldots ,B_{i}=\left[\begin{array}{c} b_{i}(1)\\ b_{i}(2)\\ b_{i}(3)\\ \ldots \\ b_{i}(2K) \end{array}\right],\ldots ,$$

where $B_{i}$ (*i *= 1,2, …) is a certain radiation source signal type to be recognized.

Assume that the knowledge base between the radiation source signal type and the signal subtle features based on part of the samples is as follows:

2.16 $$C_{1}=\left[\begin{array}{c} c_{1}(1)\\ c_{1}(2)\\ c_{1}(3)\\ \ldots \\ c_{1}(2K) \end{array}\right],\;\;C_{2}=\left[\begin{array}{c} c_{2}(1)\\ c_{2}(2)\\ c_{2}(3)\\ \ldots \\ c_{2}(2K) \end{array}\right]\ldots ,C_{j}=\left[\begin{array}{c} c_{j}(1)\\ c_{j}(2)\\ c_{j}(3)\\ \ldots \\ c_{j}(2K) \end{array}\right],\ldots ,$$

where $C_{j}$ (*j *= 1,2, …) is a known radiation source signal type and $c_{j}$ (*j *= 1,2, …) is a certain feature parameter.

For ρ∈(0,1),

2.17 $$\xi (b_{i}(k),c_{j}(k))=\frac{\min_{j}\min_{k}|b_{i}(k)-c_{j}(k)|+\rho \cdot \max_{j}\max_{k}|b_{i}(k)-c_{j}(k)|}{\left|b_{i}(k)-c_{j}(k)|+\rho \cdot \max_{j}\max_{k}|b_{i}(k)-c_{j}(k)\right|}$$

and

2.18 $$\xi (B_{i},C_{j})=\frac{1}{2K}\sum_{k=1}^{2K}\xi (b_{i}(k),c_{j}(k)),\;j=1,2,\ldots ,$$

where ρ is the distinguishing coefficient; $\xi (b_{i}(k),c_{j}(k))$ is the grey relation coefficient of the *k*th feature parameter for $B_{i}$ and $C_{j}$; and $\xi (B_{i},C_{j})$ is the grey relation degree for $B_{i}$ and $C_{j}$. Hereafter, $B_{i}$ is categorized to a certain radiation source signal type where the maximal $\xi (B_{i},C_{j})$ (*j *= 1,2, …,) is calculated.

### 2.5. Proposed approach

**Figure 1.**
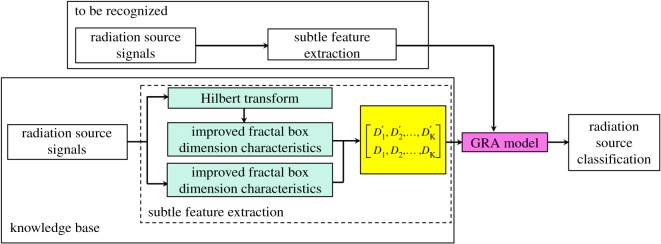
Radiation source signal recognition based on signal subtle feature extraction.

In summary, the process of the proposed method for radiation source signal recognition is as follows, and the flow chart is illustrated in figure 1.

Step 1: A variety of target radiation source signals are sampled, to establish the knowledge base.
Step 2: The subtle feature vectors are extracted from the sample knowledge base through the dual improved generalized fractal box-counting dimension eigenvectors.
Step 3: The sample knowledge base for GRA is established based on the signal symptom (i.e. the extracted subtle feature vectors $\left[D_{1}^{\prime },D_{2}^{\prime },\ldots ,D_{K}^{\prime },\;D_{1},D_{2},\ldots ,D_{K}\right]$) and the signal pattern (i.e. the known radiation source signal type).
Step 4: The subtle feature vectors extracted from the radiation source signals to be identified are input into the GRA, and the recognition results are output.

## 3. Application and analysis

In order to verify the performance of the proposed dual improved generalized fractal box-counting dimension eigenvector algorithm, 11 different radiation source signals were used to test the recognition effect, compared with the traditional fractal box-counting dimension and the single improved generalized fractal box-counting dimension. In this paper, the radiation source signals for testing are from the Case Western Reserve University Bearing Data Center. The database has been a standard dataset for testing the effectiveness of the feature extraction algorithm and the pattern recognition algorithm. In addition, the sampled signals of the database are full of random mechanical noise, which makes the test closer to the real situation. The motor drive end rotor is supported by a test bearing, where a single point of failure is set through discharge machining. The radiation source signals of the bearing vibration data used for the analysis are obtained under a motor speed of 1797 revolutions per minute and load of 0 horsepower. An accelerometer is installed on the motor drive end housing with a bandwidth of up to 5000 Hz, and the vibration data for the test bearing under different fault patterns are collected by a recorder as the radiation source signals, in which the sampling frequency is 12 kHz. The fault types contain the outer race fault, the inner race fault, and the ball fault, and the fault diameters, i.e. the fault severities, are 28 mils (1 mil = 0.0254 mm), 21 mils, 14 mils and 7 mils. In total, 11 types of radiation source signals of bearing vibration data in different fault categories and with different fault severities were analysed, as seen in table 1. Each data sample is made up of 2048 time-series points. Each of the 550 data samples is different with different random mechanical noise. Among these 550 data samples, 110 data samples were chosen randomly for the establishment of the knowledge base, with the remaining 440 data samples taken as the testing data samples.

**Table 1.** Description of the experimental dataset.

fault condition	fault diameter (mils)	the number of base samples	the number of testing samples	label of classification
normal	0	10	40	1
inner race fault	7	10	40	2
	14	10	40	3
	21	10	40	4
	28	10	40	5
ball fault	7	10	40	6
	14	10	40	7
	28	10	40	8
outer race fault	7	10	40	9
	14	10	40	10
	21	10	40	11

**Figure 2.**
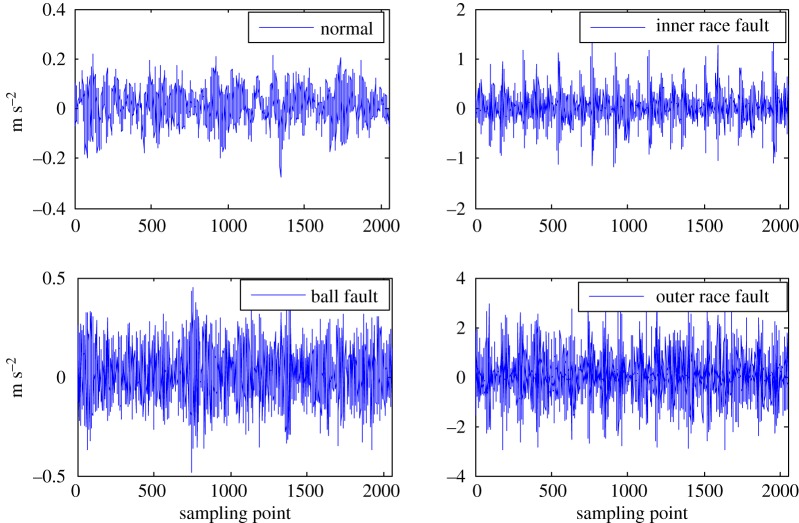
The radiation source signals of the rolling bearing normal operating conditions and various fault conditions with a fault diameter of 7 mils.

**Figure 3.**
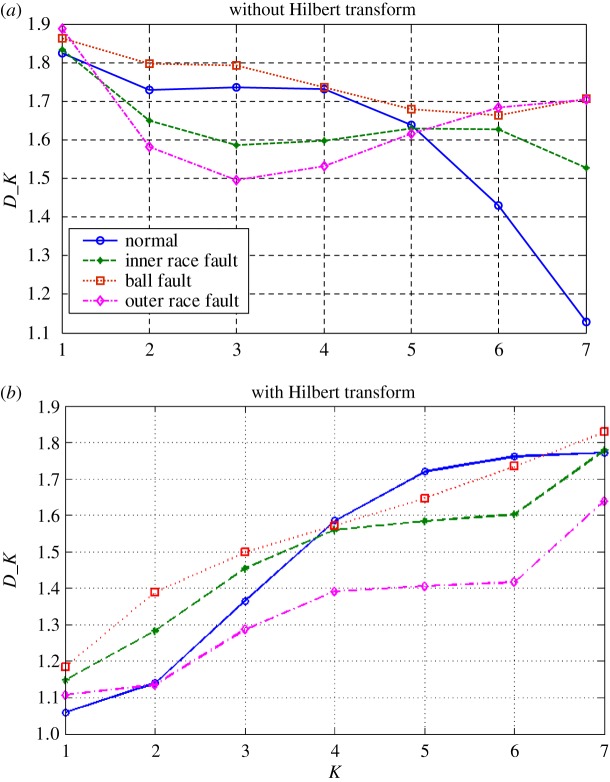
Improved generalized fractal box-counting dimensions of a randomly sample from the radiation source signals of the bearing normal conditions and different fault conditions with fault size 7 mils, (*a*) without the Hilbert transform and (*b*) with the Hilbert transform.

**Figure 4.**
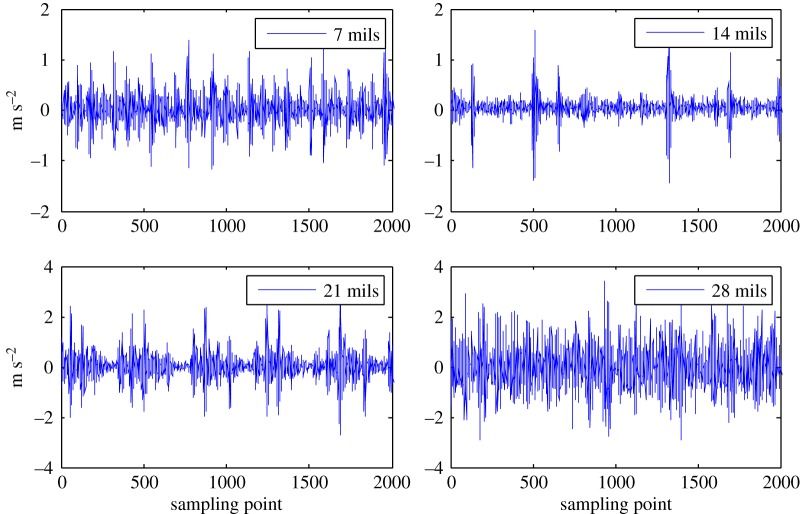
The radiation source signals of the bearing inner race fault conditions with various severities.

**Figure 5.**
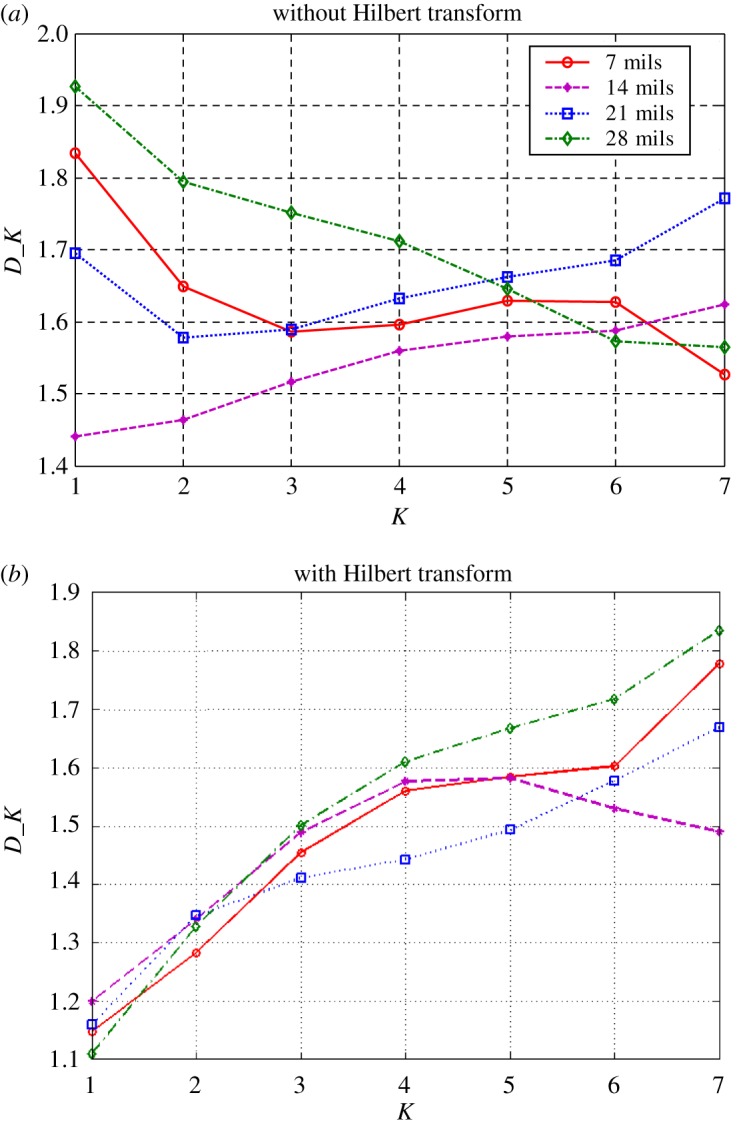
Improved generalized fractal box-counting dimensions of a randomly chosen sample from the radiation source signals of the bearing inner race fault condition with different levels of severity, (*a*) without the Hilbert transform and (*b*) with the Hilbert transform.

The subtle feature vectors extracted from the radiation source signals of the rolling bearing normal operating conditions and different fault conditions with a 7 mils fault diameter (seen in figure 2) based on the dual improved generalized fractal box-counting dimension eigenvectors are shown in figure 3. And the subtle feature vectors extracted from the radiation source signals of the bearing inner race fault condition with various severities (seen in figure 4) based on the dual improved generalized box-counting dimension eigenvectors are shown in figure 5.

From tables 2 and 3, it is can be seen that the features extracted by the traditional box-counting dimension method with and without the Hilbert transform are both limited, and the distances between the different types of radiation source signals are close, and are not easily classified by a pattern recognition technique.

**Table 2.** Traditional fractal box-counting dimensions with and without the Hilbert transform of a randomly chosen sample from radiation source signals of the bearing under normal conditions and under different fault conditions with fault size 7 mils.

signals	normal	inner race fault	ball fault	outer race fault
traditional box-counting dimension without Hilbert transform	1.5718	1.6173	1.7511	1.6000
traditional box-counting dimension with Hilbert transform	1.0640	1.1542	1.1733	1.0884

**Table 3.** Traditional box-counting dimensions of a randomly chosen sample with and without the Hilbert transform from radiation source signals of the bearing inner race fault condition with different levels of severity.

signals	7 mils	14 mils	21 mils	28 mils
traditional box-counting dimension without Hilbert transform	1.6173	1.5795	1.6356	1.6491
traditional box-counting dimension with Hilbert transform	1.1542	1.1818	1.1502	1.1096

From figures 3 and 5, it is interesting to see that the dominant subtle feature vectors $\left[{D^{\prime }}_{1},{D^{\prime }}_{2},\ldots ,{D^{\prime }}_{K},\;D_{1},D_{2},\ldots ,D_{K}\right]$ extracted from the radiation source signals of the rolling element bearing vibration signals with different fault types and severities through the dual improved generalized fractal box-counting dimension eigenvectors show apparent differences, in particular for the extracted improved fractal box-counting dimension characteristics $\left[D_{1},D_{2},\ldots ,D_{K}\right]$. The sample knowledge base for GRA is established based on the signal symptom (i.e. the extracted subtle feature vectors $\left[{D^{\prime }}_{1},{D^{\prime }}_{2},\ldots ,{D^{\prime }}_{K},\;D_{1},D_{2},\ldots ,D_{K}\right]$) and the signal pattern (i.e. the known radiation source signal types). The subtle feature vectors $\left[{D^{\prime }}_{1},{D^{\prime }}_{2},\ldots ,{D^{\prime }}_{K},\;D_{1},D_{2},\ldots ,D_{K}\right]$ extracted based on the radiation source signals of the testing rolling bearing vibration signals to be identified are input to GRA, and the recognition results are output, as shown in tables 4–6.

**Table 4.** The identification results with the traditional fractal box-counting dimension and the single improved generalized fractal box-counting dimension by GRA.

		number of misclassified samples		testing accuracy (%)	
label of classification	number of testing samples	traditional	improved	traditional	improved
1	40	18	0	55	100
2	40	8	0	80	100
3	40	20	4	50	90
4	40	18	0	55	100
5	40	14	0	65	100
6	40	22	4	45	90
7	40	31	0	22.5	100
8	40	32	4	20	90
9	40	22	0	45	100
10	40	31	0	22.5	100
11	40	16	4	60	90
in total	440	232	16	47.2727	96.3636

**Table 5.** The recognition results with the single fractal box-counting dimension eigenvector and the dual improved generalized fractal box-counting dimension eigenvectors by GRA.

		number of misclassified samples		testing accuracy (%)	
label of classification	number of testing samples	single	dual	single	dual
1	40	0	0	100	100
2	40	0	0	100	100
3	40	4	0	90	100
4	40	0	0	100	100
5	40	0	0	100	100
6	40	4	0	90	100
7	40	0	5	100	87.5
8	40	4	0	90	100
9	40	0	0	100	100
10	40	0	0	100	100
11	40	4	0	90	100
in total	440	16	5	96.3636	98.86

**Table 6.** The recognition results by the proposed method compared with results from [23] and [25]. Note: The approach of [23] is based on multifractal theory for extracting feature vectors and a GRA for achieving pattern recognition intelligently using the extracted feature vectors. The approach of [25] is based on a four-dimensional feature extraction algorithm using entropy and Holder coefficient theories for extracting feature vectors and a GRA for achieving pattern recognition intelligently using the extracted feature vectors. In our previous works, such as [23,25], we have fully compared the recognition results with the existing feature extraction algorithm (such as entropy theory, Holder coefficient theory and multifractal theory) and a pattern recognition algorithm (such as the feed-forward back-propagation neural network, the SVM, and the adaptive GRA) in the same topic. And at this stage, we propose the improved algorithm based on our previous works [23,25] to improve the signal subtle feature extraction performance based on the dual improved fractal box-counting dimension eigenvectors.

		number of misclassified samples			testing accuracy (%)		
label of classification	number of testing samples	[23]	[25]	proposed	[23]	[25]	proposed
1	40	0	0	0	100	100	100
2	40	0	0	0	100	100	100
3	40	0	2	1	100	95	100
4	40	3	0	0	92.5	100	100
5	40	0	0	0	100	100	100
6	40	2	3	2	95	92.5	100
7	40	3	0	3	92.5	100	87.5
8	40	3	4	0	92.5	90	100
9	40	0	0	0	100	100	100
10	40	0	3	0	100	92.5	100
11	40	4	0	1	90	100	100
in total	440	15	12	7	96.59	96.9697	98.86

From table 4, compared with the traditional fractal box-counting dimension algorithm, the single improved fractal box-counting dimension algorithm can better reflect the signal subtle distribution characteristics under the different reconstruction phase space and has a better recognition effect.

From table 5 and table 6, the recognition results of the proposed method illustrate that the total recognition success rate can reach 98.86%, which shows a certain improvement in the recognition accuracy after the application of the dual improved fractal box-counting dimension eigenvectors in the signal subtle feature extraction compared with the methods from [23] and [25]. The time cost of the proposed approach using a laptop computer with a 4.0 GHz dual processor for one test case is only 0.027 s and the time consumption of the proposed approach is encouraging, which shows good real-time performance.

## 4. Conclusion

Fractal dimension is an important tool to describe the complexity of a fractal body. In this paper, the fractal box-counting dimension algorithm in fractal theory is improved, and a dual improved generalized fractal box-counting dimension eigenvector algorithm is proposed. The proposed algorithm can effectively characterize the fractal box-counting dimensionality of radiation source signals under different reconstructed phase space. The experimental results show that, compared with the traditional fractal box-counting dimension algorithm and the single improved fractal box-counting dimension algorithm, the proposed dual improved fractal box-counting dimension algorithm can better extract the signal subtle distribution characteristics under different reconstruction phase space, and has a better recognition effect with good real-time performance. The experimental study has illustrated the following.

(1) The proposed method can accurately and effectively recognize different radiation source signal types.
(2) The recognition results using the proposed method show that the total recognition success rate can reach 98.86%.
(3) The proposed method can improve the recognition performance compared with the existing pattern recognition methods, and the time consumption of the proposed approach is encouraging.

In future work, other types of radiation source signals can be further used for testing the effectiveness of the proposed approach.

## Data accessibility

The rolling bearing vibration signals for testing are from the Case Western Reserve University Bearing Data Center (http://csegroups.case.edu/bearingdatacenter/pages/download-data-file (accessed 11 October 2017)).
